# Surgical Considerations in Treating Central Nervous System Lymphomas: A Case Series of 11 Patients

**DOI:** 10.3390/curroncol31110491

**Published:** 2024-10-25

**Authors:** Corneliu Toader, Adrian Vasile Dumitru, Mugurel Petrinel Radoi, Luca-Andrei Glavan, Milena-Monica Ilie, Razvan-Adrian Covache-Busuioc, Vlad Buica, Antonio-Daniel Corlatescu, Horia-Petre Costin, Carla Crivoi, Alexandru Vlad Ciurea

**Affiliations:** 1 Department of Neurosurgery, “Carol Davila” University of Medicine and Pharmacy, 020021 Bucharest, Romania; corneliu.toader@umfcd.ro (C.T.); luca-andrei.glavan0720@stud.umfcd.ro (L.-A.G.); milena-monica.ilie0720@stud.umfcd.ro (M.-M.I.); razvan-adrian.covache-busuioc0720@stud.umfcd.ro (R.-A.C.-B.); vlad.buica0720@stud.umfcd.ro (V.B.); antonio.corlatescu0920@stud.umfcd.ro (A.-D.C.); horia-petre.costin0720@stud.umfcd.ro (H.-P.C.); prof.avciurea@gmail.com (A.V.C.); 2 Department of Vascular Neurosurgery, National Institute of Neurovascular Disease, 077160 Bucharest, Romania; 3 Department of Pathology, University Emergency Hospital, 050098 Bucharest, Romania; 4 Department of Computer Science, Faculty of Mathematics and Computer Science, University of Bucharest, 010014 Bucharest, Romania; crivoicarla02@gmail.com; 5 Neurosurgery Department and Scientific, Sanador Clinical Hospital, 010991 Bucharest, Romania

**Keywords:** lymphoma, tumor resection, surgical treatment, CNS, DLBCL

## Abstract

In this retrospective unicentric study, we analyzed the medical records of 11 patients who were surgically treated for CNS lymphoma, both primary and secondary, between 2009 and 2024. Given the rarity of CNS lymphomas and their diverse signs and symptoms based on tumoral location, our aim was to describe key aspects, such as clinical presentations and surgical management. A possible relationship between obesity and CNS lymphoma progression was investigated through an analysis of previous study findings. The literature suggests a wide spectrum of manifestations, from nausea and headaches to loss of equilibrium and speech impairment. A predominance of unsystematized balance disorders and epileptic seizures were affirmed. Notably, as emerged from our study, aphasia was a particularly interesting neurological symptom due to its rarity in the clinical features of CNSL. Other significant factors, such as tumor localization and perioperative phases, were thoroughly investigated, with the latter highlighted by an illustrative case report. Additionally, a literature review was included, comprising nine recent retrospective studies on the efficacy of surgical resection for patients diagnosed with PCNSL.

## 1. Introduction

According to the WHO’s nosology, central nervous system lymphomas (CNSLs) are a type of hematolymphoid tumors, including primary diffuse large B cell lymphoma of the CNS, immunodeficiency-associated CNS lymphoma, lymphomatoid granulomatosis and intravascular large B cell lymphoma [1]. Most commonly, CNSL manifests as a diffuse large B cell lymphoma (DLBCL) (in 90% of cases), followed by T cell (2%), Burkitt’s, lymphoblastic, and low-grade lymphomas [2].

Central nervous system lymphoma (CNSL) can be either primary or secondary. The former type (PCNSL) has its origins in the brain parenchyma, leptomeninges, pachymeninges, spinal cord, or eyes, without any traces of lymphoma outside of the CNS [3]. This rare and aggressive extranodal malignancy accounts for approximately 3% of all CNS tumors and has a dismal outcome, with a median overall survival of approximately 3 years [4,5]. In secondary CNSL (SCNSL), the presence of the tumor is the consequence of a disseminating systemic lymphoma or a relapse following treatment. As a result, the tumor can exist concomitantly with the systemic involvement or it can be isolated as a solitary brain mass [6]. For patients already diagnosed with DLBLC, the risk of developing SCNSL is about 5%, but it goes up to 15% for differing co-existing physiological aspects [6].

For PCNSL, the incidence rate is 0.47 per 100,000 persons-years, with a significantly higher rate in the male population (0.55 per 100,000 p-y) compared to the female population (0.39 per 100,000 p-y) [7]. Although the exact cause for PCNSL is unknown, factors such as immunodeficiency, post-transplant immunosuppression, and advanced age significantly augment the probability of onset [7]. More precisely, infection with human immunodeficiency virus (HIV) increases the risk of PCNSL by 3600-fold in comparison with the general population [8].

Patients have diverse clinical presentations depending on the tumoral localization, but B-symptoms are rarely associated with PCNS lymphoma [9,10].

Although a stereotactic biopsy is considered the gold standard for CNSL diagnosis, the role of gross or subtotal tumoral resection remains ambiguous in the literature, with no clear recommendations [11,12]. Surgery becomes an option in the case of single lesions in patients with low perioperative morbidity or in the case of acute serious symptoms [4,13]. Whole brain radiation therapy (WBRT) alone was abandoned decades ago in favor of more beneficial therapeutical approaches [13]. Multimodal chemotherapy with high-dose methotrexate (HD-MTX) and Rituximab—a monoclonal antibody against the CD20 antigen—are the principal agents used for treating both PCNSL and SCNSL [14]. The median overall survival was greater for HD-MTX alone (25 to 55 months) compared to WBRT alone (12 to 18 months) [13]. Usually after chemotherapy, two additional strategies are considered, autologous stem cell transplantation (ASCT) and WBRT, but there has been no consensus on the optimal regimen [14].

Given the limited data on the efficacy of surgical interventions in such cases, we aimed to present the cases of 11 patients who underwent gross total resection (GTR) of CNSL to document their symptoms and outcomes and to contribute to the growing body of knowledge in this field.

## 2. Materials and Methods

This article analyzes 11 cases of CNS lymphoma treated surgically with gross total resection (GTR) from 2009 to 2024 at the Department of Neurosurgery, National Institute of Neurology and Neurovascular Diseases in Bucharest, Romania. Data on the diagnosis, treatment, and follow-up were retrospectively collected, focusing on specific variables during the preoperative, intraoperative, and postoperative phases. Patients were either referred for surgery by a multidisciplinary team following a previous diagnosis of systemic lymphoma or presented with cerebral symptoms to the Neurology Department. In every case, gross tumor resections were performed, and diagnoses were confirmed through histopathological and immunochemistry reports. Follow-up was conducted either in the inpatient or outpatient ward, with an average duration of 44.27 months. The longest follow-up period was 96 months, while the shortest was 2 months. Moreover, postoperative complications were discussed, and the number of reoperations was acknowledged.

This research adheres to the main principles in the Declaration of Helsinki and received approval from the Ethics Committee of the National Institute of Neurology and Neurovascular Diseases in Bucharest, Romania (Ethical Review Board of National Institute of Neurology and Neurovascular Diseases; approval number 6466). Clinical data, including assessments of BMI and arterial hypertension, Glasgow Coma Scale scores, and the presence of epileptic seizures and balance disorders, were extracted from the relevant files. The processing of all data was conducted in compliance with current GDPR guidelines, and informed consent was obtained from all patients included in this study.

Statistical analysis and figure plotting were conducted using Python version 3.10, developed by the Python Software Foundation, located at 9450 SW Gemini Dr., ECM# 90772, Beaverton, OR 97008, USA. The analysis involved the use of Python libraries, such as pandas, numpy, seaborn, and matplotlib.

## 3. Results

**Figure 1:**
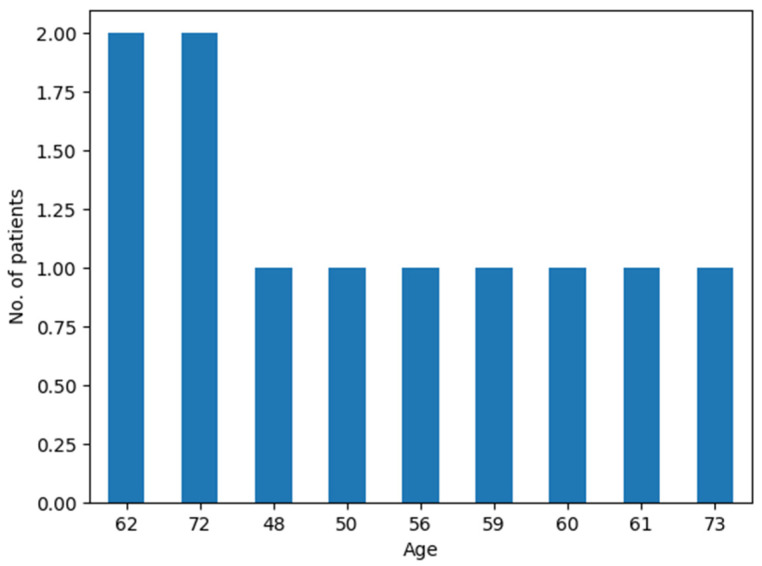
Age distribution in the CNS lymphoma patient cohort.

In this study, which included a total of 11 patients (10 with SCNSL and 1 with PCNSL), males comprised 63.6% (*n* = 7) of all confirmed CNSL cases, while females accounted for 36.4% (*n* = 4) of cases. Only one male had PCNSL. The male-to-female ratio for SCNSL was 1.5:1 (six males to four females). The median age for undergoing surgery was 61 years, with an average age of 61.3 years and an age range between 48 and 72 years (Figure 1).

**Figure 2:**
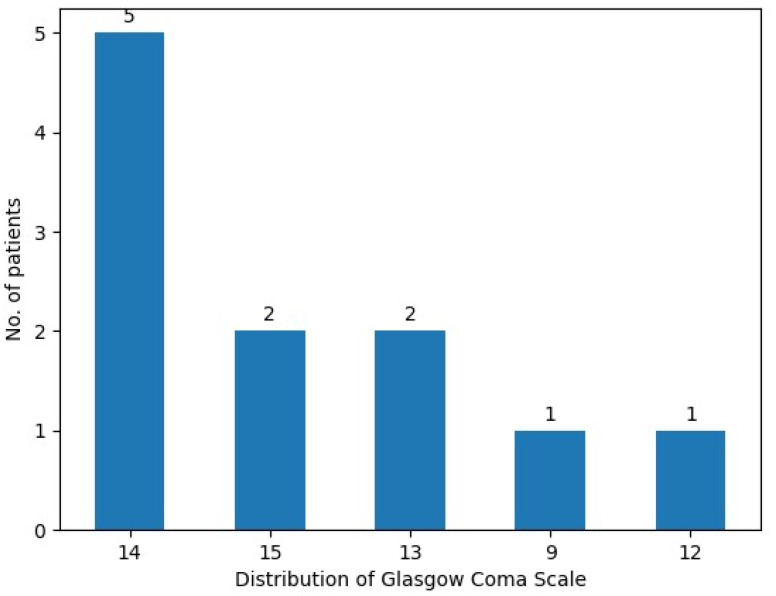
Distribution of Glasgow Coma Scale scores in the CNS lymphoma patient cohort.

At admission, two patients (18.1%) presented with a Glasgow Coma Scale (GCS) score of 15, while five patients (45.4%) measured a GCS score of 14, and another two patients (18.1%) presented with a score of 13. Moderate acute traumatic brain injury was indicated by GCS scores of 12 and 9 in the other two patients assessed (Figure 2).

General physical examinations included the calculation of the body mass index and the assessment of blood pressure. Nine patients (81.8%) were not classified as obese, having BMIs lower than 30 kg/m^2^. One (9%) was categorized under obesity class II, and another one (9%) was classified under obesity class III.

Eight patients (72.7%) had normal blood pressure, two of them (18.1%) presented with stage 2 hypertension, and one (9%) presented with stage 1 hypertension.

The medical history and a neurological examination showed that only four (36.3%) patients had unsystematized balance disorders, while three patients (27.2%) reported experiencing epileptic seizures.

Three patients (27.2%) received neo-adjuvant therapy (chemotherapy and radiotherapy combined) for PCNSL or systemic lymphoma with central nervous system metastases.

**Figure 3:**
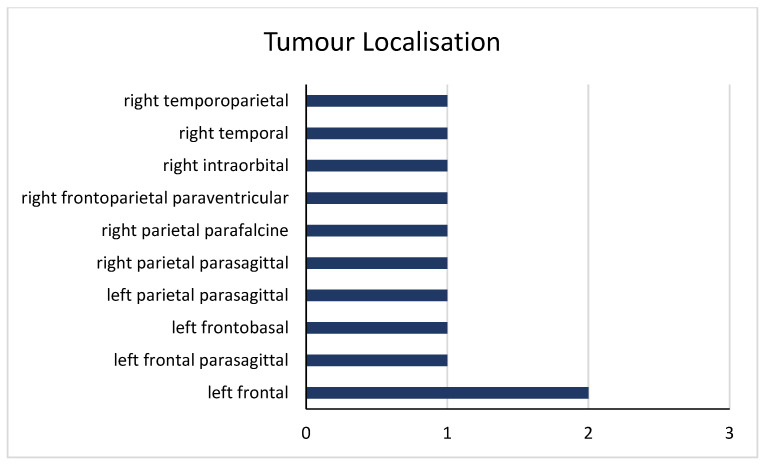
Distribution of tumoral locations in the CNS lymphoma patient cohort.

Medical imaging and an intraoperative examination focused on two tumoral characteristics: localization and dimension (maximum diameter). The dimensions of the tumor ranged from 25 to 70 mm, with a mean diameter of 40.9 mm and a median diameter of 40 mm. Regarding tumoral mapping, four tumors were located in the frontal lobe, three were located in the parietal lobe, and one spanned both the frontal and parietal lobes. Additionally, one was situated in the temporal and parietal region, one was located in the temporal lobe, and another was intraorbital (Figure 3). A pattern of distribution seems to arise from the data set, with a site predilection for the frontal lobe (36.3% of all tumors).

The microscopic diagnoses were established post-tumoral excision, with histopathology and immunochemistry findings revealing that 90.9% of all CNS lymphomas were of the DLBCL type. Only one patient was classified under non-B cell lymphoma subtypes, either T cell or NK cell lymphoma, although definitive laboratory results were not obtained.

**Figure 4:**
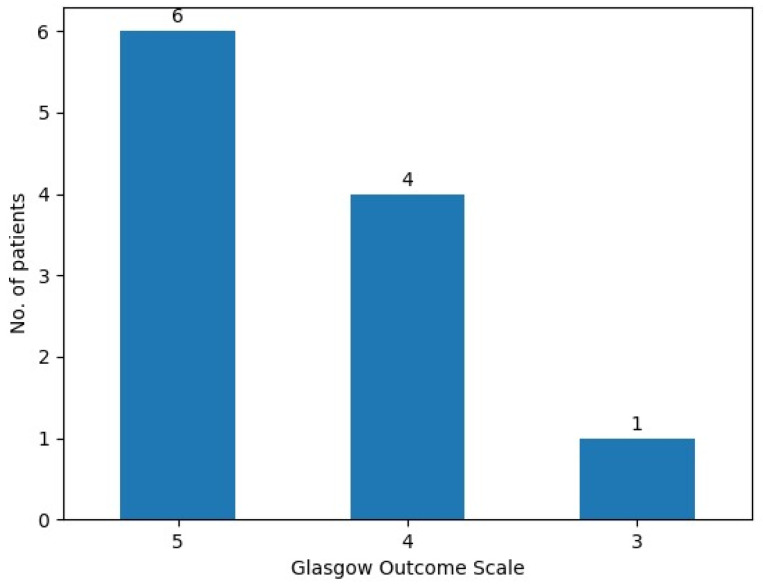
Glasgow Outcome Scale scores.

The Glasgow Outcome Scale was used to evaluate patient evolution before hospital discharge, indicating an improvement in the general condition of our patients, with six patients (54.5%) having a maximum score of five, four (36.3%) having a score of 4—showing moderate disability but independence in daily activities—and only one patient (9%) with a score of three—indicating severe disability and total dependence (Figure 4).

Postoperative surveillance consisted of regular appointments with the following healthcare team: neurosurgeon, oncologist, and radiologist. In three cases (27.2%) there has been documented evidence of tumor recurrence. The remaining eight (72.7%) consistently showed no clinical or imaging evidence suggestive of CNS lymphoma relapse. The three patients suffering from CNSL relapse were either unfit for surgery or refused a second surgical intervention.

**Figure 5:**
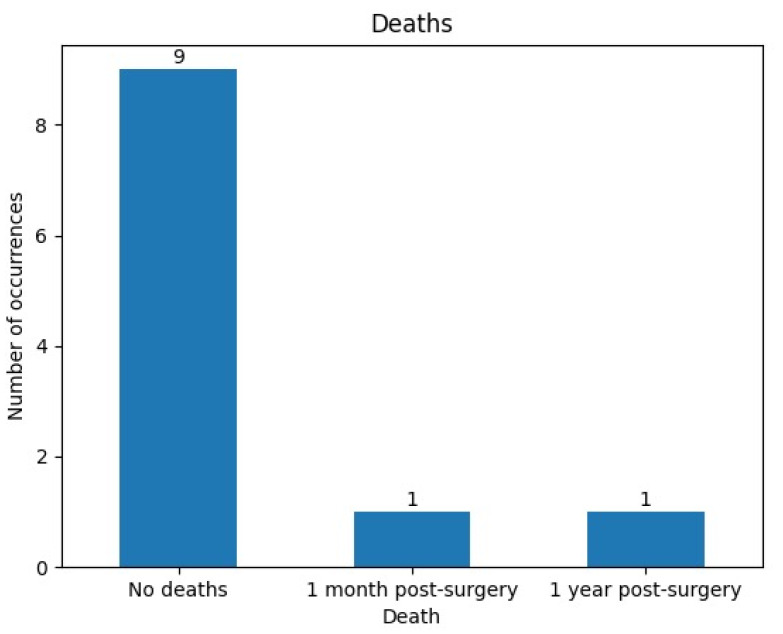
Number of deaths after surgical resection in the CNS lymphoma patient cohort.

Despite excellent postoperative management, two (18.1%) of our patients died within one year following surgery, endorsing the well-known aggressive nature of CNS lymphoma (Figure 5).

## 4. Case Presentation

### 4.1. Patient Profile

A 73-year-old male with a complex medical history, including multiple vascular and pulmonary conditions, presents a challenging clinical picture involving neurological and hematologic pathologies.

### 4.2. First Admission

The patient was initially admitted with a diagnosis of a right temporoparietal brain tumor, suspected to be lymphoma, and recurrent Jacksonian seizures affecting the left hemibody. His past medical history included diffuse non-Hodgkin small B cell lymphoma treated with chemotherapy, COPD, sequelae of pulmonary micronodules, moderate normochromic and macrocytic anemia, acquired immunodeficiency, cerebral and systemic atherosclerosis, cerebral abiotrophy syndrome, benign prostatic hypertrophy, and a right cortical renal cyst.

Upon admission, the patient exhibited marked somnolence, polypnea, and obnubilation. He scored 13 on the Glasgow Coma Scale (GCS) and had recurrent left hemibody Jacksonian seizures, gait and orthostatic disturbances, left hemiparesis (predominantly brachial), and memory and attention deficits. Additionally, he had incontinence issues.

**Figure 6:**
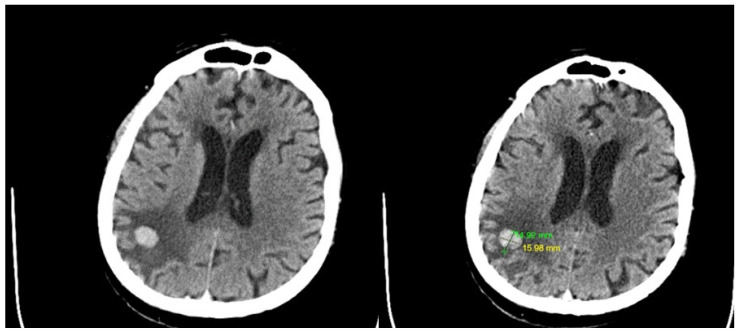
Preoperative contrast-enhanced CT scan revealed a dense, iodophilic nodule measuring approximately 16/14 mm in the right parietal subcortical area associated with digitiform edema in the adjacent white matter. No other pathological contrast enhancements were observed within the brain parenchyma. Leukoaraiosis was present, but there were no intra- or pericerebral hemorrhagic accumulations.

The preoperative contrast-enhanced CT scan showed a dense, iodophilic nodule, approximately 16/14 mm in size, located in the right parietal subcortical area, accompanied by digitiform edema in the surrounding white matter (Figure 6).

Under general anesthesia, the patient underwent right temporoparietal craniotomy and tumor resection. Postoperatively, there was significant neurological improvement, including the resolution of left hemiparesis and cessation of partial motor seizures. The patient was discharged conscious, coherent, afebrile, ambulatory with assistance, with resolved left hemiparesis and seizures, and stable cardio-respiratory and hemodynamic status. 

**Figure 7:**
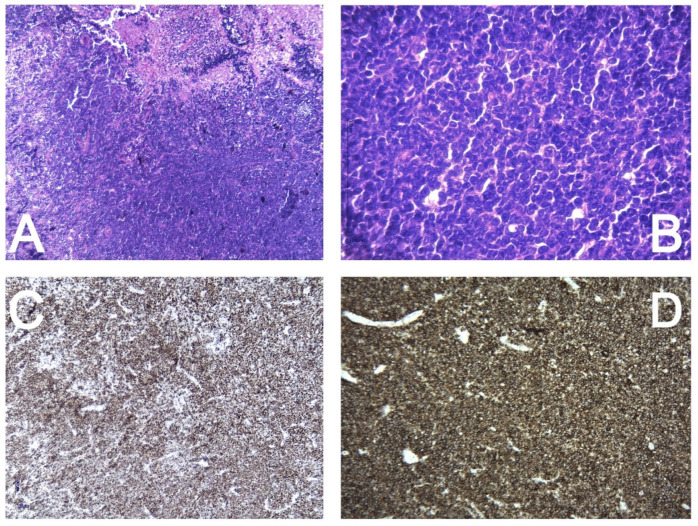
Histopathology and immunohistochemistry report. Diffuse, with high cellular proliferation and extensive necrosis (4×—(**A**)). The tumor cells are large with irregular, atypical nuclei and distinct nucleoli, resembling centroblasts (40×—(**B**)). Ki67—diffusely positive in 90% of the tumor cells (**C**). CD20—strongly and diffusely positive in the tumor cells (**D**). CD34, CD10, and BCL6—negative expression in the tumor cells. MUM1—diffuse positive nuclear expression in the tumor cells. CD5—moderate positivity in most of the tumor proliferation. BCL2—diffusely positive in the tumor cells.

The histopathology and immunohistochemistry report revealed a diffuse tumor with high cellular proliferation and extensive necrosis, as seen under low magnification (4×—A). The tumor cells were large, with irregular, atypical nuclei and prominent nucleoli, resembling centroblasts at higher magnification (40×—B). Ki67 was diffusely positive in 90% of the tumor cells (C), indicating significant proliferation. CD20 was strongly and diffusely positive in the tumor cells (D), while CD34, CD10, and BCL6 showed no expression. MUM1 exhibited diffuse positive nuclear expression, and CD5 showed moderate positivity in most of the tumor proliferation. BCL2 was diffusely positive throughout the tumor cells. Based on this immunophenotypic profile, the diagnosis of diffuse large B cell lymphoma was established (Figure 7).

### 4.3. Second Admission

**Figure 8:**
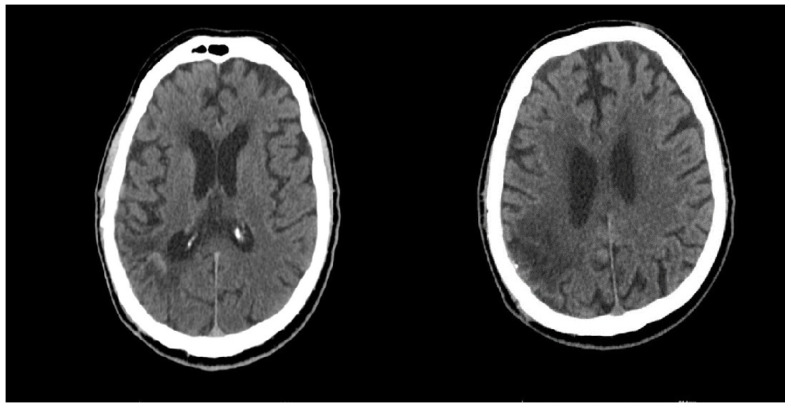
One year post-surgery, a contrast-enhanced CT shows no sign of tumor recurrence.

One year later, the patient was readmitted with progressive neurological deterioration, including bilateral blindness, the inability to walk, and variable epileptic seizures. a Contrast-enhanced CT shows no sign of tumor recurrence and no new vascular abnormalities were noted (Figure 8).

His vital signs were stable. Neurologically, the patient presented with bilateral blindness, spastic left hemiparesis, orofacial clonus, crural myoclonus (predominantly left-sided), and mild to moderate dysarthria. The laboratory findings indicated mild normochromic normocytic anemia, hepatic cytolysis, and normal renal function. An EEG showed diffuse theta activity without epileptiform features, while an MRI did not reveal new lesions. Given the clinical and biochemical profile, Phenytoin was discontinued due to hepatic cytolysis, with increased doses of Levetiracetam and the addition of Clonazepam. Low-molecular weight heparin was administered for DVT prophylaxis. The patient showed slight improvement in seizure control but remained neurologically impaired.

The patient was discharged hemodynamically stable, transported home with ambulance support, and required continuous care.

## 5. Discussion

An exhaustive data collection from The Central Brain Tumor Registry of the United States (CBTRUS) between 2013 and 2017 concluded that, in general, men are more likely to develop malignant CNS tumors [15]. In particular, primary CNS lymphomas correlated with a male-to-female ratio of 1.2:1 (6:5) [15]. By contrast, secondary CNSL did not benefit from extensive gender statistics, warranting future exploration. In our study, the male-to-female ratio for SCNSL was 1.5:1 (six males to four females), while the only patient with PCNSL was male.

According to the literature, the systemic involvement present in secondary CNS lymphoma (SCNSL) typically results in poorer outcomes compared to primary CNS lymphoma (PCNSL), which originates de novo within the central nervous system [16]. In a very important study conducted by Ferreri et al., several factors emerged as reliable predictors of survival for patients diagnosed with PCNSL [17]. Among other parameters, the study concluded that an age of more than 60 and the involvement of specific CNS areas (periventricular regions, basal ganglia, brainstem, and/or cerebellum) independently predicted worse survival outcomes [17].

To this day, in the literature, there is a paucity of comprehensive correlations between SCNSL and obesity. A 2013 study by Patel et al. significantly contributed to understanding the relationship between obesity and malignant tumors, suggesting that obesity might play a crucial role in lymphomagenesis, potentially increasing the incidence of non-Hodgkin lymphomas, especially DLBCL [18]. As part of the routine physical examination upon admission, the medical team measured the body mass index (BMI) of each patient. In our study, we observed that both obese patients in our cohort were diagnosed with secondary central nervous system lymphoma (SCNSL) of the DLBCL type. Despite receiving appropriate oncological care post-surgery, one patient with stage 2 obesity unfortunately died one year after the surgical intervention.

Due to its potential to appear in various forms and areas within the central nervous system, a “constellation of symptoms” describes, with accuracy, the clinical presentation of CNS lymphomas. Moreover, as first described in 1996 by Alderson et al., transient lesions called “sentinel lesions” can also raise difficulties in the diagnosis process of PCNSL because of their inconstant appearance on imaging tests and non-specific histopathological characteristics [19].

In light of the previous literature findings, clinical presentations of CNSL vary from mild symptoms, such as nausea, dizziness, and headaches, to serious neurocognitive impairments, such as epileptic seizures, aphasia, pyramidal syndrome, and balance disorders [20,21]. In PCNSL, because the tumor is confined within the central nervous system, weight loss, fever, and night sweats only occur in exceptional cases [22]. Notably, two patients had expressive aphasia upon admission, with both tumoral processes being located in the frontal lobe.

A systematic review conducted by Aboubakr et al. in 2023 that analyzed 21 previous studies on seizure occurrence in PCNSL concluded that a third of all patients reported experiencing comitial seizures, mostly at the moment of hospital admission [23]. However, incomplete and inconclusive information during follow-up limits interpretation regarding causality or relevance. In our study, at the time of admission, 27.2% (a total of three) of patients reported experiencing epileptic seizures with the following variable semiological presentations: Jacksonian partial seizures or generalized tonic-clonic seizures. All three were prescribed oral antiepileptics after hospital discharge.

Both gait and balance disorders are noticed at admission in patients later diagnosed with CNSL [24]. Medical history and physical examinations revealed that 36.3% (a total of four patients) of our subjects presented with unsystematized balance disorders. Their specific tumor localizations were as follows: two in the left frontal lobe, one in the right parietal lobe, and one in the right temporoparietal region.

Unlike the case of PCNSL, in which numerous pivotal studies about pharmacological treatment options have been conducted, SCNSL did not benefit from great scientific attention and lacks extensive data [6,25,26]. The 2022 guidelines from the British Society of Hematology primarily base their chemoradiotherapy recommendations for SCNSL on single-arm phase II trials [27]. A study conducted by Weller et al. in 2012 challenged the long-standing belief that advised against gross or subtotal tumor resection in patients diagnosed with PCNSL, encouraging practitioners to consider surgical management for patients with PCNSL when this type of invasive approach seems safe, such as in cases with single lesions [28,29]. The European Association of Neuro-Oncology (EANO) guidelines from 2023 recommend that patients diagnosed with PCNSL through stereotactic biopsy should undergo high-dose methotrexate-based chemotherapy. However, the role of cytoreductive surgery versus biopsy in PCNSL management remains a subject of debate due to the lack of prospective studies comparing survival outcomes and morbidity [30].

Patients were admitted to our clinic under two main scenarios: following a multidisciplinary team decision for combined chemoradiotherapy and gross total resection or presenting with neurological symptoms that required surgical intervention, without a prior diagnosis of systemic lymphoma. To differentiate between PCNSL and SCNSL, all patients underwent extensive imaging evaluations, including thoracic, abdominal, and pelvic MRI or CT scans. Among the 11 patients in our cohort, 10 were diagnosed with SCNSL, while only 1 patient was confirmed to have PCNSL. The majority of cases in our study were secondary manifestations of systemic disease. It is widely accepted in the literature that patients later diagnosed with systemic lymphoma often present to the hospital with non-specific symptoms due to the slow progression of the disease, which can range from dyspepsia (in the case of gastrointestinal lymphomas) to comitial seizures (when neurological involvement is present) [31,32]. These patients exhibited a range of neurological symptoms prior to their CNSL diagnosis, including dysphasia, aphasia, frontal lobe syndrome, and seizures (both generalized and partial). Importantly, the onset of these symptoms occurred within a window of less than one year preceding the formal diagnosis of CNSL. All patients underwent gross total resection of their solitary lesions, aiming to maximize tumor removal while minimizing residual disease. Neurological improvement was observed in all patients post-surgery, with symptoms such as dysphasia and frontal lobe syndrome disappearance, and the frequency of seizures reduced.

Table 1 provides a comprehensive overview of nine recent cohort studies examining the impact of surgical resection on OS, PFS, and postoperative functionality in patients with PCNSL. The findings suggest that surgical intervention, particularly when combined with other treatment modalities, may confer survival benefits in select patient populations.

The retrospective nature, heterogeneous sample sizes, and diverse or sometimes unspecified surgical approaches are limitations that underscore the need for further research to definitively establish the role of surgical resection in PCNSL management.

## 6. Conclusions

Our case series adds to a growing body of literature on the surgical management of primary and secondary CNS lymphomas, providing meaningful insights into tumoral localization, links with obesity, clinical manifestations, and surgical management. Interestingly, a predilection for the frontal lobe was observed (36.3%) among our patients. Acknowledging that hesitance dominated experts’ opinions on tumor resection, we observed a 27.2% recurrence rate and an 18.1% death rate for this rare and aggressive tumor. Additionally, a literature review of nine recent cohort studies from 2018 to 2023 analyzing the potential benefits of surgical resection in patients with PCNSL lesions was included. While the outcomes are promising, current findings still support the previous recommendations for the rigorous assessment of risks and benefits when considering surgery for CNS lymphomas, except in cases of life-threatening manifestations.

## Figures and Tables

**Table 1:** OS = overall survival, PFS = progression-free survival, GTR = gross total resection, STR = subtotal resection.

Article Title	Authors and Reference	Country of Data Collection	Study Design	Principal Objective	Number of Patients Undergoing Surgical Resection	Type of Surgical Intervention	Conclusions
Real-World Impact of Surgical Excision on Overall Survival in Primary Central Nervous System Lymphoma	Deng et al. (2020) [33]	USA	retrospective	To investigate the association of surgery with PCNSL prognosis by comparing SO between patients who underwent surgical resection and those who did not	851	STR and GTR	Combining surgical excision with chemotherapy yields longer OS than chemotherapy alone, suggesting the benefits of multimodal treatment.
The role of surgical resection in primary central nervous system lymphoma: a single-center retrospective analysis of 70 patients	Wu et al. (2021) [34]	China	retrospective	To evaluate the effect of surgical resection and stereotactic biopsy on the complication rate, PFS, and OS of patients diagnosed with PCNSL	28	Resection (not specified)	Although the outcomes for patients with PCNSL in the cohort remain poor, surgical resection may significantly improve OS and PFS compared to stereotactic biopsy in a subset of patients.
Clinical Characteristics, Surgical Outcomes, and Prognostic Factors of Intracranial Primary Central Nervous System Lymphoma	Ouyang et al. (2020) [35]	China	retrospective	To analyze the outcomes and complications of surgical treatment for intracranial PCNSL	71	STR and GTR	For intracranial PCNSL, surgical excision can improve PFS, but not OS.
Resection of primary central nervous system lymphoma: impact of patient selection on overall survival	Schellekes et al. (2021) [36]	Israel and Milan	retrospective	To investigate the impact of the resection of a solitary lesion on the survival of PCNSL patients by comparing outcomes between those who underwent resection and those who had a needle biopsy	36	STR and GTR	In a subgroup of patients with superficial tumors, patients who underwent surgical resection had significantly longer OS than those who underwent needle biopsy.
Primary central nervous system lymphoma in China: a single-center retrospective analysis of 167 cases	Yuan et al. (2019) [37]	China	retrospective	To evaluate the potential prognostic factors for OS and PFS in patients with PCNSL	62	GTR	Gross total resection was found to be an independent favorable prognostic factor for OS.
The role of surgery in primary central nervous system lymphomas	Villalonga et al. (2018) [38]	Argentina	retrospective	To compare survival among patients with PCNSL who underwent biopsy versus surgical resection	18	STR and GTR	Patients who underwent surgical resection had significantly longer survival than those who had a biopsy alone, with a survival difference of almost one-and-a-half years.
Functional Outcome and Overall Survival in Patients with Primary or Secondary CNS Lymphoma after Surgical Resection vs. Biopsy	Staub-Bartelt et al. (2023) [20]	Germany	retrospective	To compare the postoperative functional status and OS of patients with PCNSL who underwent resection with those who underwent either stereotactic or open biopsy	35	STR and GTR	Patients who underwent surgical resection experienced longer survival outcomes and more favorable postoperative functionality.
The Safety of Resection for Primary Central Nervous System Lymphoma: A single institution retrospective analysis	Cloney et al. (2018) [29]	USA	retrospective	To analyze the safety of surgical resection for patients with PCNSL	58	Resection (not specified)	Postoperative complication rates following surgical resection were found to be similar to those observed in other intracranial neoplasms.
Evaluation of Memorial Sloan-Kettering Cancer Center and International Extranodal Lymphoma Study Group prognostic scoring systems to predict Overall Survival in intracranial Primary CNS lymphoma	Jahr et al. (2018) [39]	Norway	retrospective	To evaluate the safety of surgical resection for patients with PCNSL	32	Resection (not specified)	The complication rates identified after the resection of PCNSL were comparable and considered acceptable relative to those reported in studies of other CNS tumors.

## Data Availability

Available upon reasonable request from the corresponding author.
